# Supplementary material for: Changes in food pricing and availability on the Navajo Nation following a 2% tax on unhealthy foods: The Healthy Diné Nation Act of 2014
Source: PLoS One. 2021 Sep 2;16(9):e0256683. doi: 10.1371/journal.pone.0256683 (PMC8412325; doi:10.1371/journal.pone.0256683)
Supplement: S1 Appendix — (DOCX) [file pone.0256683.s001.docx]

**S1 Appendix. Percentage of Navajo and border town stores offering healthier items in 2013 and 2019, n=71**

|  | **2013** | |  | **2019** | |  |
| --- | --- | --- | --- | --- | --- | --- |
| **Healthy item** | **Navajo stores**  **(n=51)** | **Border Town stores**  **(n=20)** |  | **Navajo stores**  **(n=51)** | **Border Town stores (n=20)** | **p-value (comparing NN to Border in 2019)** |
| Any fruit | 82% | 65% |  | 82% | 80% | 1.0 |
| 3+ types of fruit | 65% | 50% |  | 69% | 70% | 1.0 |
| Apples | 77% | 60% |  | 80% | 65% | 0.15 |
| Oranges | 73% | 50% |  | 73% | 24% | 0.29 |
| Bananas | 59% | 65% |  | 61% | 75% | 0.17 |
| Any vegetables | 88% | 60% |  | 78% | 70% | 0.66 |
| 3+ types of vegetables | 73% | 60% |  | 71% | 50% | 0.18 |
| Tomatoes | 61% | 60% |  | 65% | 45% | 0.10 |
| Corn | 16% | 40% |  | 28% | 25% | 0.79 |
| Celery | 41% | 45% |  | 49% | 40% | 0.65 |
| Lettuce | 73% | 60% |  | 59% | 50% | 0.51 |
| Potatoes | 73% | 55% |  | 65% | 40% | 0.04 |
| Squash | 24% | 45% |  | 25% | 30% | 0.77 |
| Bottled Water | 98% | 100% |  | 100% | 100% | 1.0 |
| Healthy/low calorie beverages | 100% | 100% |  | 100% | 100% | 1.0 |
| 100% juice | 96% | 95% |  | 98% | 100% | 1.0 |
| Canned vegetables | 82% | 85% |  | 84% | 80% | 0.97 |
| Canned fruit | 78% | 53% |  | 80% | 60% | 0.55 |
| > 4 healthy snacks | 75% | 70% |  | 77% | 90% | 0.34 |
| Baked chips | 41% | 60% |  | 51% | 85% | 0.03 |
