# Supplementary material for: Changes in food pricing and availability on the Navajo Nation following a 2% tax on unhealthy foods: The Healthy Diné Nation Act of 2014
Source: PLoS One. 2021 Sep 2;16(9):e0256683. doi: 10.1371/journal.pone.0256683 (PMC8412325; doi:10.1371/journal.pone.0256683)
Supplement: S2 Appendix — (DOCX) [file pone.0256683.s002.docx]

**S2 Appendix. Inflation-adjusted average price of food items in Navajo and border town grocery and convenience Stores, 2013 and 2019**

|  | **Navajo grocery stores (n= 8)** | **Border grocery stores (n=8)** | **Navajo convenience stores (n= 24)** | **Border convenience stores (n=11)** |  | **Navajo grocery stores (n= 8)** | **Border grocery stores (n=8)** | **Navajo convenience stores (n= 24)** | **Border convenience stores (n=11)** |
| --- | --- | --- | --- | --- | --- | --- | --- | --- | --- |
| **Healthier food item** | **2013** | | | |  | **Average price 2019 (in 2013 dollars)** | | | |
| **Water** | 1 gallon water | | 1 gallon water | |  | 1 gallon water | | 1 gallon water | |
|  | $1.04 (n=8) | $0.89 (n=7) | $1.71 (n=15) | $1.83 (n=10) |  | $0.99 (n=7) | $0.82 (n=7) | $1.52 (n=21) | $1.66 (n=11) |
| **Diet Coke** | 12 pack | | 20 oz | |  | 12 pack | | 20 oz | |
|  | $5.78 (n=8) | $4.81 (n=8) | $1.54 (n=22) | $1.60 (n=11) |  | $5.20 (n=8) | $4.48 (n=8) | $1.64 (n=21) | $1.77 (n=11) |
| **J00% Orange Juice** | Minute Maid, 59 oz | | Minute Maid, 15.2 oz | |  | Minute Maid, 59 oz | | Minute Maid, 15.2 oz | |
|  | $3.92 (n=8) | $2.94 (n=8) | $1.85 (n=18) | $1.79 (n=11) |  | $3.04 (n=7) | $2.87 (n=8) | $1.78 (n=21) | $1.82 (n=9) |
| **Healthy Chips** | Baked Lays (6 oz) | | Baked Lays (single serving, 2 oz) | |  | Baked Lays (6 oz) | | Baked Lays (single serving, 2 oz) | |
|  | $3.49 (n=4) | $3.62 (n=3) | $1.69 (n=7) | $2.09 (n=4) |  | $2.89 (n=6) | $2.99 (n=8) | $2.01 (n=11) | $1.95 (n=8) |
| **Less healthy item** | **Average price 2013** | | | |  | **Average price 2019 (in 2013 dollars)** | | | |
| **Soda (Coke)** | 12 pack | | 20 oz | |  | 12 pack | | 20 oz | |
|  | $5.78 (n=8) | $4.81 (n=8) | $1.54 (n=22) | $1.60 (n=11) |  | $5.20 (n=8) | $4.48 (n=8) | $1.64 (n=21) | $1.77 (n=11) |
| **Juice drink** | Juice drink, 59 oz | | Juice drink, 15.2 oz | |  | Juice drink, 59 oz | | Juice drink, 15.2 oz | |
|  | $2.84 (n=8) | $1.36 (n=7) | $1.84 (n=17) | $1.74 (n=9) |  | $2.24 (n=7) | $1.76 (n=8) | $1.75 (n=17) | $1.81 (n=11) |
| **Chips** | Classic Lays (10 oz) | | Classic Lays (10 oz) | |  | Classic Lays (10 oz) | | Classic Lays (10 oz) | |
|  | $3.23 (n=7) | $3.31 (n=3) | $2.97 (n=22) | $2.76 (n=6) |  | $3.63 (n=8) | $2.85 (n=7) | $2.08 (n=17) | 2.91 (n=11) |
