# Supplementary material for: Changes in food pricing and availability on the Navajo Nation following a 2% tax on unhealthy foods: The Healthy Diné Nation Act of 2014
Source: PLoS One. 2021 Sep 2;16(9):e0256683. doi: 10.1371/journal.pone.0256683 (PMC8412325; doi:10.1371/journal.pone.0256683)
Supplement: Attachment — Submitted filename: May 20 PlosOne Responses to reviewer comments sss.docx [file pone.0256683.s003.docx]

**Article title:** *Changes in food pricing and availability on the Navajo Nation following a 2% tax on unhealthy foods: the Healthy Diné Nation Act of 2014*

**Responses to reviewer/editorial comments.**

**Editor comments:**

*Thank you for submitting your manuscript to PLOS ONE. After careful consideration, we feel that it has merit but does not fully meet PLOS ONE’s publication criteria as it currently stands. Therefore, we invite you to submit a revised version of the manuscript that addresses the points raised during the review process.*

*Please focus in particular on the comments related to the gap identified in the introduction and including some methodologic details regarding the border towns and display of prices on the shelves.*

**Overall Response:**

Thank you for these comments and thoughtful review of the manuscript overall. We feel it has been greatly strengthened as a result of the feedback. Notably, we have adjusted the introduction to more appropriately focus on changes in the Navajo Nation food environment and comparison to border town stores added clarification throughout to several methodological issues (and also added these as limitations in the discussion as appropriate). In addition, we have included how pricing data was collected, clarified issues regarding item pricing per piece and per pound, included additional details about the NEMS survey categories and clarified store promotion of the HDNA legislation. Detailed responses to reviewer comments are outlined below.

**Reviewer comments:**

**Reviewer #1:** *Thank you for the opportunity to review this manuscript. As the authors note, no prior studies have assessed changes in the food store environment after the implementation of the HDNA, which is a topic of interest to health researchers and policymakers, among others. The paper is well-written and synthesizes existing research on the subject clearly.

My primary concerns relate to how price data was collected, particularly in convenience stores, in which prices may or may not be listed on the shelf. If they were all posted, then please specify that in the text, and if not, it would be helpful to know from what percentage of stores the prices were obtained from the store owner (or by purchasing the items).*

**Response:** Thank you for this comment. We have clarified that data collection was based on observational surveys and marked prices, not purchases. If pricing information was missing, store staff was inquired. The primary reason for this approach ­­was to follow the same procedures as were implemented as in 2013 to allow for direct comparison in pricing between 2013 and 2019. We have clarified this in the text and also added this as a limitation in the discussion section.

***Comment:*** *Additionally, it is unclear whether the sales tax was included in the prices, which is an important consideration given that this analysis looks at changes in price and the waiver of sales tax on the price of healthy foods is a key component of HDNA. Other, more minor suggestions are described below.*

**Response:** Sales tax was not included since the price information was based on marked prices. This has now also been clarified and added as a limitation to the discussion section.

***Comment:*** *Ln 35: suggest “offered” in place of “sold” for clarity, since data was collected on product availability, and can’t tell us about sales.*

**Response:** This change was made.

***Comment:*** *Ln 56: “Tribal” is capitalized here, but not elsewhere (e.g., line 80). Suggest using whichever convention is preferred consistently throughout*.

**Response:** We made it lowercase to be consistent throughout.

***Comment:*** *Ln 79: Philadelphia’s tax is technically a “sweetened beverage tax”, rather than a sugar-sweetened beverage tax since it applies to artificially sweetened beverages as well. Suggest choosing a different example city or dropping the “sugar-“.*
**Response:** Thank you! We dropped the word sugar.

***Comment:*** *Line 97: add “would” after “healthy foods”*
**Response:** This change was made. However, we subsequently removed the sentences as we want to be cognizant we are not analyzing sales data.

***Comment:*** *Ln 98: What do you mean by “regional variability”? Did you stratify by region?*
**Response:** Apologies this was indeed unclear. We intended to compare the on-reservation to border town changes and have removed the text referring to regional variability (there are 5 regions on the Navajo Nation, but since the HDNA was applied across the entire Navajo Nation, so we did not compare HDNA implementation across Navajo Nation regions).

***Comment:*** *Ln 113: Sentence is a bit confusing- was the list updated in 2019 or was it updated annually?*
**Response:** Apologies, this was confusing. The list was updated annually as part of general research team maintenance of the store database. The 2019 update was the list used for the follow-up data collection, which is why we referred to it specifically. We tried to clarify the sentence.

***Comment:*** *Lns 145-150: Were prices listed on shelf tags (or the products themselves) or were prices obtained by asking the store owner/manager, or a combination? Did you collect data about whether prices were displayed in the store or not? Also, if prices were listed on the shelf tag, was sales tax included, not included, or was it unknown? I think this is highly relevant given the content of the HDNA law.*

**Response:** This is indeed important context. Prices were based on observational data and shelf prices using the NEMS survey to match the procedures implemented in 2013. Store staff was asked if pricing information was missing (the frequency was not formally recorded, but it was rare). We have aimed to clarify this throughout the manuscript and added this as a limitation.

***Comment:*** *Lns 161-164: What do you mean by the number/variety of healthy food options and the ratio of healthful/unhealthful options? Is this based on all items available in the store or a specific set of items from the NEMS tool?*
**Response:** The number and variety of items was indeed based on the NEMS tool, which has 10 sections such as milk, breads & tortillas, fruits & vegetables, meats and hot dogs, canned foods etc. There are subsections under each section for specific individual types of foods; for example, 3 types of fruits and 6 types of vegetables are specifically listed and checked: apples, bananas, oranges, tomatoes, celery, lettuce, corn, squash, potatoes. It was also assessed if healthier versions of certain foods were available (i.e. low fat milk, lean ground beef, 100% juice). In addition, the tool specifies the number of items under each section (for example, for fruits, vegetables, chips etc.), with answer options being 0,1,2,3,4,5 and 6 or more. The data was summarized for the individual foods and the overall number of foods available under each section. Finally, foods were categorized as healthful or unhealthful and the ratio of these was calculated by dividing the total number of available food types in each category. We have added more detail to the methods section to clarify the procedures and ratio.

***Comment:*** *Ln 164: How did stores demonstrate that they had implemented HDNA? Was this measured based on presence of a sign in the stores?*
**Response:** Indeed, this was noted based on presence of a sign or other promotional information (flyer etc.) in the store. This has been added to clarify.

***Comment:*** *Lns 168-169: This is confusing. Are prices not presented unless the produce item was priced per item? Were they included in the analysis? This seems problematic if grocery stores typically price produce by weight.*
**Response:** Apologies, we agree this was confusing. The NEMS survey indicates to record the price per cheapest unit being sold. If a store offered a type of produce both per-piece and per-pound, only the per-pound price was recorded as it almost always is the cheapest per unit price. Because it is challenging to aggregate data across per-piece or per-pound pricing methods, comparisons between 2013 and 2019 pricing were made separately for per piece and per pound pricing. Indeed, grocery stores typically only sold produce per pound. For example, in the full cohort, 17 stores in 2013 offered apples only per pound and 18 in 2019, and the average price was compared between these stores for the cost of apples per pound. We have attempted to clarify the abovementioned sentences to reflect this approach more clearly.

***Comment:*** *Lns 180-181: Was drop-out/closure differential by location (on or off-reservation)? Also, what proportion of stores that dropped out (or closed) were grocery stores?*
**Response:** Of the 32 stores that were closed/declined participation or had missing geocode data, 88% were on the Navajo Nation, 12% in border towns, 81% were convenience stores, 16% grocery stores and 3% other. This information has been added to the manuscript.

***Comment:*** *Ln 199: Sentence should make it clear that the p-value for fruit was non-significant (unless there is a typo).*
**Response:** We added the letters ns. to indicate the p-value was indeed not significant. This was incidentally not a typo, as the p-value was 1.0 rounded up as percentages were nearly identical for convenience stores offering fruits on the Navajo Nation (82%) and in bordertowns (80%). We have also added .ns for the other comparisons in the same paragraph that were not statistically significant.

***Comment:*** *Ln 210: add “more” before “likely.”*
**Response:** Thank you, this change was made.

***Comment:*** *Ln 219: “Bordertown” sounds like a single place when capitalized and in the singular.*
**Response:** We agree and revised this to read: *“P-value comparing convenience stores on the Navajo Nation to border towns.”*

***Comment:*** *Lns 222-3: Is this out of all stores or out of Navajo stores only? Did no bordertown stores sell traditional foods?*
**Response:** Unfortunately traditional foods were only assessed in the Navajo stores in 2013. Thus, we only reported traditional food availability in the Navajo grocery and convenience stores in 2019 as well to facilitate comparability between samples. We have added this clarification to the methods section.

***Comment:*** *Ln 287: “wasn’t” should be “was not.”*
**Response:** This change was made.

***Comment:*** *Ln 312: It is unclear who “they” are- is this store owners?*
**Response:** Indeed, these are the store managers. This was changed.

***Comment:*** *Table 2: Add total “Ns” in the header row (e.g., Navajo convenience stores, N=39)*
**Response:** The N’s were added to the header row.

***Comment:*** *Table 4: Suggest changing the title from “Trends” to “Changes”, since data is from two time points. Also, was there a reason p-values weren’t included for the Navajo store v. border store comparison?*

**Response:** We agree and Trends was changed to Changes. P-values were added to Table 4.

**Reviewer #2: Overall thoughts and summary:**
*The authors’ work is important and novel and merits publication with minor revisions.*

***Major comment:***
*• Overall, I was confused about how the authors conceptualized the link between the HDNA legislation and changes in pricing and availability of healthy and unhealthy foods in stores subject to the legislation. My impression after reading the Introduction was that the authors were exploring a potential mechanism for their previous work -- specifically that the food tax likely lead to a decrease in purchasing of unhealthy foods. However, lines 282-283 and 329-331 in the Discussion make me think otherwise. Why do the authors state that “it is not possible to determine whether changes in the food environment were due to the HDNA legislation or other factors”? If the rationale for looking at changes in pricing/availability are NOT related to the food tax, then why make it the focus of the introduction?*

**Response:** We agree this was confusing and we have changed the introduction to limit the focus on any reductions in purchasing of unhealthy foods since purchase data are not available, and instead emphasize the focus on changes in the Navajo food store environment between 2013 and 2019 and compared to border towns. Lines 282-283 were removed and lines 329-331 rephrased to indicate that modest improvements (i.e. Navajo store pricing ‘catching up’ with border stores in some cases like fruits and 100% juice, more health promotion, explicit HDNA signage etc.) suggest some extent of increased awareness, but that further study is needed to which extent these were spurred by the HDNA. In-depth interviews with store managers will take place throughout 2021 to gain further insight into these questions.

**Minor comments:
*Comment:*** *• Can you state earlier, in the methods section, why you are comparing outcomes of interest between the Navajo nation and border towns?*
**Response:** We have now stated this earlier. Our main reason was that border towns are not subject to the HDNA legislation (neither the waiver of sales tax on healthy foods nor the added 2% HDNA tax on unhealthy foods. In addition, food accessibility issues on the Navajo Nation are a key reason behind the work in general and Navajo stores being able to offer similar healthy food options at similar price points to the border towns would be an important step in improving the food environment for the Navajo people.

***Comment:*** *• Line 233: Can you first state that the cost of fresh fruits was higher in Navajo (vs. border) stores at baseline?*
**Response:** We have now added this.

***Comment:*** *• Lines 255-256 and 269-270: Can you comment further on the implications of so few stores demonstrating implementation of the HDNA legislation?*
**Response:** We have now added language clarifying that these were stores that had promotional signage explicitly showing they were implementing the HDNA. There is no requirement for the stores to advertise the HDNA implementation. As a result, it is possible that store managers simply did not prioritize adding signage or did not want to necessarily advertise they were taxing an additional 2% on unhealthy foods. We are currently conducting interviews with store managers to gain insight into questions such as these about communication of the HDNA to consumers and motivation behind adding promotional signage about the HDNA. We have added this in the text to clarify.

***Comment:*** *• Lines 324-326: Suggest adding this sensitivity analysis to the methods and results.*

**Response:** The sensitivity analysis was added to the methods and results section. Notably, results were highly similar: on average, prices were about 3 cents different between the exact matched sample and total sample (some were higher and some lower, when all values were added, average summed difference was 0.4 cents per item). The largest difference for any one item in 2019 was 12 cents for celery per pound ($1.81 in the total cohort, $1.93 in the exact matched sample). The same comparisons were still statistically significant.
