# Supplementary material for: Changes in food pricing and availability on the Navajo Nation following a 2% tax on unhealthy foods: The Healthy Diné Nation Act of 2014
Source: PLoS One. 2021 Sep 2;16(9):e0256683. doi: 10.1371/journal.pone.0256683 (PMC8412325; doi:10.1371/journal.pone.0256683)
Supplement: Attachment — Submitted filename: AUG 9 PlosOne Responses to reviewer comments.docx [file pone.0256683.s004.docx]

**Article title:** *Changes in food pricing and availability on the Navajo Nation following a 2% tax on unhealthy foods: the Healthy Diné Nation Act of 2014*

**Responses to reviewer/editorial comments.**

**Editor comments:**

*Thank you for submitting your manuscript to PLOS ONE. After careful consideration, we feel that it has merit but does not fully meet PLOS ONE’s publication criteria as it currently stands. Therefore, we invite you to submit a revised version of the manuscript that addresses the points raised during the review process.*

***Response:*** Thank you for the opportunity to further clarify the manuscript and add additional information on statistical significance. We have made the requested revisions and outlined responses to each comment below.

**Reviewer #1: Overall
Comment:** This is interesting and well-written paper on a topic that has not been published about previously. The authors’ revisions have improved the clarity of the paper, although there are still some remaining issues to be addressed. Most of my suggestions are minor, although there is one methodological question to be considered.

**Response:** Thank you for your thoughtful and detailed review. We have appreciated the opportunity for additional clarification and believe the manuscript was further refined and improved as a result.

**Comments:**
Line 30: The word “of” is missing after “the majority.”
Line 35: I suggest revising to say “While more Navajo stores offered traditional foods in 2019 (54%) compared to 2013 (38%), this trend was not statistically significant” to make the comparison clearer.

**Response:** We agree and have made these revisions.

**Comment:** Lines 73-77: My understanding is that the HDNA is a sales tax, which is applied at the point of purchase. I suggest adding an additional sentence or clause to clarify that the prices of items subject to the HDNA likely do not reflect the price increase due to the tax, which would be applied at the register (if that is correct).

**Response:** Indeed, this is correct and a good point of clarification. We have added a sentence to clarify the HDNA tax was applied at the point-of-sale at the register.

**Comment:** Line 122: Suggest deleting the word “popular.”

**Response:** The word popular has been removed.

**Comment:** Lines 169-171: Most of the comparisons you make are between the Navajo Nation stores and the border town stores at the same time point. I don’t see a problem with that; however, you do in some cases compare the difference in availability or price over time for Navajo Nation stores compared to border town stores in the text (e.g., the abstract & lines 233-235). In most cases, you don’t report on the statistical significance of the change (“The cost of fresh fruit decreased by 18% in Navajo stores, compared with 6% in border stores”), which you may want to consider doing, so that the reader knows if the change is in fact significantly different from zero, and if the change in Navajo stores is statistically significantly different from the change in border stores (difference-in-differences).

Additionally, you report in the abstract that “While more Navajo stores offered traditional foods (38% in 2013 v. 54% in 2019), this trend was not statistically significant.” The section in the paper on traditional food availability does not include statistical tests, so it is not clear how this was compared. Given that you have matched samples, if you are comparing the same group of stores (e.g., Navajo Nation stores) at two time points, I believe you should use a paired t-test for continuous variables (or a non-parametric test like the Wilcoxon signed rank test if the change in price for the item is not normally distributed) and McNemar’s test for binary variables.

**Response:** Thank you for these comments as we feel these analyses add valuable detail to the manuscript regarding statistical significance. For the pricing data, we updated the estimates for change in price and added p-values from the paired t-tests for both Navajo Nation stores and border towns. The initial comparisons averaged price changes for each fruit individually and then averaged the totals (initially, no paired comparison test was conducted). However, this did not take into account differences in sample sizes for individual fruits, so in the updated analyses, we calculated average change in price for all fruits combined (n=69 and n=75 for Navajo stores, n=9 and n=18 for border stores) and conducted t-tests. The updated analyses show a decrease of 13% in overall cost of fruit in Navajo Nation stores, from $0.88 to $0.76, which was significant p=0.029. The average cost in border town stores increased from $0.63 to $0.73, p=.519. Text in the results section and abstract were updated to reflect the changes. We also reformatted Table 3 to include the p-values for the comparison of change in average price between 2013 and 2019 for individual fruits and vegetables and also for average price per item of fruit.

Regarding traditional food availability, we added further detail to provide additional description of the *types* of traditional foods (blue corn, yellow corn, mutton or wild animals or wild plants) to align with what was presented in Figure 2. The availability of mutton, blue corn and wild plants increased from 2013 to 2019, although changes were not statistically significant. We have replaced the original text with these more detailed analyses and added the p-values for tests of significance for each individual traditional food to the section on traditional foods.

**Comment:** Line 179: missing “a” before “total”

**Response:** The ‘a’ was added..

**Comment:** Line 189: Suggest adding “all” before “stores” so that it is clear that you are talking about the full sample, not only the Navajo Nations stores. Also, 75% is substantially more than half, so you might re-word to say “Most stores (75%) had additional venues...” or something similar.

**Response:** We agree and this was changed to ‘Three quarters’ of all stores…”

**Comment:** Lines 233-235: These lines currently refer the reader to Table 3, which does not show any measure of statistical significance, nor does it display the average cost of fresh fruit overall or the difference in average cost (referenced in the abstract and the lines mentioned).
**Response:** We added the average cost of fresh fruit to the table, updated the abstract and text with this information and added p-values for the specific comparisons mentioned. None of the individual price findings were statistically significant, although the overall cost of fruit decreased significantly in Navajo Nation stores.

**Comment:** Table 4 shows the statistical significance of the difference between sign presence in Navajo stores and border towns in 2013 and again in 2019, with a column showing the magnitude of the change over time by store location (on or off reservation), but no statistical measures. The table is also missing a footnote describing what the p-values indicate.

**Response:** We conducted a regression analysis to calculate the difference-in-difference, including a time x location interaction term. The description of the regression analysis was added to the methods section and the p-values for the difference in difference and footnote were added to Table 4.

**Comment:** Line 279: The table indicates a difference of 17% for signs promoting healthy eating, not 18%.
**Response:** This was changed.

**Comment:** Line 299: If prices were measured in the same season in 2013 and 2019, shouldn’t this be a six-year period?
**Response:** This is correct and this was changed to a six-year period.

**Comment:** Line 312: Suggest checking for consistency throughout the paper for use of “Navajo Nation” v. “the Navajo Nation.”
**Response:** We have changed throughout the read the Navajo Nation.

**Comment:** Line 321: There is a typo here (“Nation Nation”).
**Response:** Thank you, this was changed.

**Comment:** Line 343: Suggest adding “including the HDNA” after “sales tax”, since the HDNA is also a type of sales tax (if this is accurate).
**Response:** Yes, this is correct and was added.

**Comment:** Line 356: Suggest deleting “likely” before “necessarily.”

**Response:** This was changed.

**Comment:** Line 360: Unclear what high-risk refers to – food security? Diet-related chronic disease?

**Response:** We revised the sentence to indicate that the high risk referred to food insecurity and food-related chronic conditions.
